# Addressing the Vaccine Hesitancy Continuum: An Audience Segmentation Analysis of American Adults Who Did Not Receive the 2009 H1N1 Vaccine

**DOI:** 10.3390/vaccines3030556

**Published:** 2015-07-15

**Authors:** Shoba Ramanadhan, Ezequiel Galarce, Ziming Xuan, Jaclyn Alexander-Molloy, Kasisomayajula Viswanath

**Affiliations:** 1 Center for Community-Based Research, Dana-Farber Cancer Institute, 450 Brookline Ave, LW 601, Boston, MA 02215, USA; E-Mails: jaclyn_alexander-molloy@dfci.harvard.edu (J.A.-M.); vish_viswanath@dfci.harvard.edu (K.V.); 2 Department of Social and Behavioral Sciences, Harvard School of Public Health, 677 Huntington Avenue, Boston, MA 02115, USA; 3 School of Public Health, University of California, Berkeley, University Hall, Rm 233, Oxford St, Berkeley, CA 94704, USA; E-Mail: galarcee@berkeley.edu; 4 Boston University School of Public Health, 801 Massachusetts Ave., Crosstown Center, Boston, MA 02118, USA; E-Mail: zxuan@bu.edu

**Keywords:** Vaccine hesitancy, audience segmentation, communication, public engagement, H1N1

## Abstract

Understanding the heterogeneity of groups along the vaccine hesitancy continuum presents an opportunity to tailor and increase the impact of public engagement efforts with these groups. Audience segmentation can support these goals, as demonstrated here in the context of the 2009 H1N1 vaccine. In March 2010, we surveyed 1569 respondents, drawn from a nationally representative sample of American adults, with oversampling of racial/ethnic minorities and persons living below the United States Federal Poverty Level. Guided by the Structural Influence Model, we assessed knowledge, attitudes, and behaviors related to H1N1; communication outcomes; and social determinants. Among those who did not receive the vaccine (*n* = 1166), cluster analysis identified three vaccine-hesitant subgroups. Disengaged Skeptics (67%) were furthest from vaccine acceptance, with low levels of concern and engagement. The Informed Unconvinced (19%) were sophisticated consumers of media and health information who may not have been reached with information to motivate vaccination. The Open to Persuasion cluster (14%) had the highest levels of concern and motivation and may have required engagement about vaccination broadly. There were significant sociodemographic differences between groups. This analysis highlights the potential to use segmentation techniques to identify subgroups on the vaccine hesitancy continuum and tailor public engagement efforts accordingly.

## 1. Introduction

The contribution of vaccines to reduced morbidity and mortality is one of the great public health success stories [1,2]. Broadly, compliance with vaccine recommendations is high in high-income countries and vaccination rates have improved over recent decades in low- and middle-income countries [3]. Yet this general trend in vaccine success masks underlying complexities in political, logistical, cultural, economic, and social constraints, resulting in unequal immunization rates across populations [4,5]. Vaccination faces challenges from a variety of fronts and, as a result, vaccination rates are uneven and questions regarding vaccination are increasing across the globe [6,7,8]. This leads to frequent outbreaks of childhood and adult infectious diseases that could be easily prevented, such as recent measles outbreaks in Wales, United Kingdom and California, United States and endemic polio in Nigeria [9,10,11].

Although individuals were traditionally considered to be “pro”- or “anti”- vaccination, such a dichotomous characterization masks variation and results in missed opportunities to develop customized communication strategies and reach large groups who may be open to vaccination. The diversity of groups along the continuum of beliefs and attitudes towards vaccination prompts attention to “vaccine hesitancy” [12,13]. The vaccine-hesitant category includes individuals who are concerned about specific vaccines or vaccination schedules; reject all vaccines, sometimes due to religious or philosophical reasons; do not have specific issues with vaccines, but are concerned based on media and interpersonal communications related to vaccines; have low demand for vaccines because they have not seen the impact of vaccine-preventable diseases; have concerns with trust in/legitimacy of institutions involved with vaccination; and/or are motivated by desires to be informed consumers and advocates for their families’ health [8,12,13,14,15,16,17]. The drivers of vaccine hesitancy are diverse, including contextual influences (such as culture, healthcare systems, political structures, *etc.*); individual and social group influences (such as norms, beliefs, *etc.*); and vaccine- and vaccination-specific issues (such as supply and delivery, role of healthcare professionals, costs, health risk and benefit, vaccine exemption policies, and introduction of new vaccines or formulations) [13].

It is vital to understand the diversity of vaccine-hesitant subgroups to support public health goals of large-scale vaccine uptake. Those who are hesitant, but do not reject all vaccines, are of particular interest from a public health standpoint. This subset is a larger group than those who completely reject vaccines and may be more amenable to changing beliefs and behaviors as they tend to seek out and engage with information about vaccines [15]. The study of vaccine hesitancy is vital both to address and stem challenges to the successful public health intervention strategy of controlling vaccine-preventable disease, but also in the context of emergency preparedness. Here, we take the example of the H1N1 pandemic response of 2009–2010 in the United States.

The 2009 H1N1 influenza virus (referred to as “H1N1” throughout) emerged and began to spread rapidly across the United States in April of that year. This resulted in a wide-scale emergency response in the United States, which emphasized activation of public health preparedness systems as well as education of the general public regarding this strain of influenza, risks of contracting the virus, and key prevention strategies, including vaccination [18,19]. At the outset of the pandemic, approximately half of the public reported they would receive the vaccine and 59%–70% of parents expected to vaccinate their children, according to national polls [18]. However, by April 2010, only 24% of adults had received the vaccination, well below the rates of regular seasonal influenza vaccination [20]. This low uptake rate was attributed to a combination of factors, including limited initial vaccine availability, concerns about the safety of what was perceived to be a newly developed vaccine, decreasing perception of susceptibility, and decreasing perception of disease severity. Interestingly, members of the public suggested that the vaccination decision was a trade-off between the potential risk of illness and the potential risk of the vaccine [18]. Vaccine uptake and its psychosocial antecedents were unevenly distributed across population subgroups [21]. It is useful to study the vaccine hesitancy continuum in the context of a single vaccine/disease given that attitudes and perceptions are often vaccine- and disease-specific [22]. In the case of vaccines perceived by the public to be novel—like H1N1—hesitancy may include concerns about safety, side effects, disease severity, the rapid vaccine development process, and financial incentives for vaccine producers [18,23].

Despite public concerns, governments and public health agencies must act quickly and effectively to stop pandemic influenza strains from having massive impacts across the globe. Beyond vaccine production and distribution, a major tool in their arsenal is communication. Yet, officials and authorities often utilize a top-down, expert-led approach, with a generic, one-size-fits-all approach [24]. Such approaches are unlikely to lead to behavior change across population sub-groups. A multi-pronged effort, involving interactive dialogue, interpersonal and mass media communication, and public engagement is likely necessary to support individuals in making decisions about vaccines that are evidence-informed [15,22,25]. Such an effort requires understanding the diverse audiences along the vaccine hesitancy continuum for whom communication/engagement campaigns must be created.

**Figure 1:**
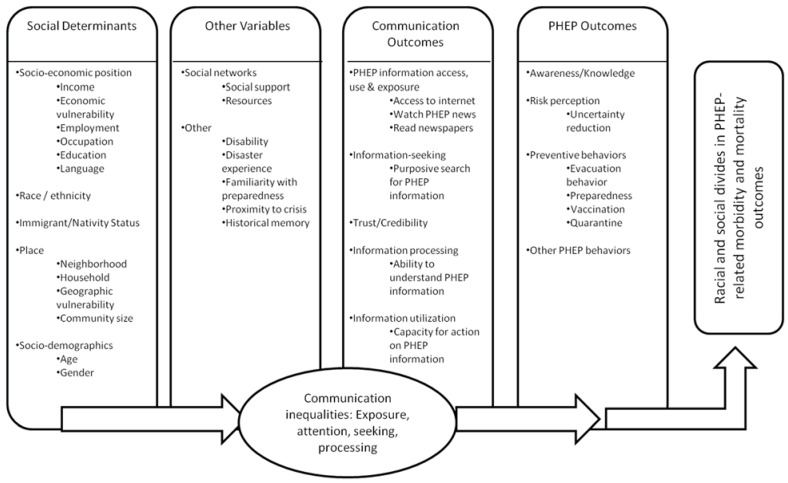
Structural Influence Model of PHEP Communications.

Audience segmentation is a useful method to identify population subgroups and determine effective ways to promote behavior change among these groups. Dividing large, heterogeneous groups into meaningful, more homogeneous subgroups is expected to allow health communication professionals to determine which groups to target and how best to do so. To identify key population subgroups, the population is divided based on patterned responses across key variables, such as attitudes, beliefs, and behaviors [26,27,28]. A theory-driven approach for variable selection ensures a targeted selection of these variables. We ground this investigation in the Structural Influence Model (SIM), adapted for Public Health Emergency Preparedness (PHEP) as shown below in Figure 1.

The SIM posits that inequalities in communication (such as differential exposure to information for population subgroups) mediate the relationship between social determinants (such as socioeconomic position, race/ethnicity, or other factors highlighted in the figure) and health outcomes, thus serving as one potential explanation for health disparities. In other words, health disparities can be understood in part as a function of how (1) structural determinants such as socioeconomic position and (2) moderating mechanisms such as social networks lead to (3) differential communication outcomes, such as access to and use of information channels, attention to health content, recall of information, and capacity to act on relevant information, which in turn drive (4) awareness/knowledge, risk perception, preventive behaviors, and other emergency preparedness behaviors [29]. For this segmentation analysis, we applied the framework working from the endpoints (PHEP outcomes) backwards to understand the link between audience segments and key theoretical drivers of outcomes of interest.

Utilizing the SIM as a theoretical framework, we developed three research questions for this study. First, what are the key audience segments among individuals who did not receive H1N1 vaccination based on key PHEP outcomes (awareness/knowledge, risk perception, and preventive behaviors)? Second, were there significant differences between audience segments in communication outcomes (information access, use, and exposure; information-seeking; and trust/credibility)? Third, were there significant differences between audience segments in social determinants (SEP, race/ethnicity, immigrant/nativity status, and place)?

## 2. Results

We drew from a nationally representative sample of adults in the United States, with oversampling of minority ethnic/racial groups and those living under the United States Federal Poverty Level (*n* = 1569, response rate = 66/3%). Details regarding the full sample can be found elsewhere [21]. We restricted the analysis to individuals who had not received the H1N1 vaccine and had complete data for the clustering variables (*n* = 1166). As described in the Materials and Methods section in greater detail, we conducted a cluster analysis using as input variables the “PHEP outcomes” section of the theoretical framework: knowledge of H1N1, risk perception, vaccine safety, protective behavior against H1N1, and seasonal flu vaccine receipt.

The demographic profile of the sample is presented in Table 1. The 1166 individuals ranged in age from 18 to 94 years, with an average of 44 years. About 44% had annual incomes below 100% of the United States Federal Poverty Level. About half of the sample had education levels of high school degree or less. The sample included 41% individuals who identified as White, non-Hispanic, 44% as Hispanic, and almost 10% as African-American, non-Hispanic.

**Table 1:** Socio-demographic profile of sample (*n* = 1166).

Characteristics	Number	Percent
Poverty level		
Below 100%	514	44.1
Age (years)		
18–24	120	10.3
25–34	261	22.4
35–44	251	21.5
45–54	231	19.8
55–64	175	15.0
65–74	103	8.8
75+	25	2.1
Income		
Less than $20,000	484	41.5
$20,000–$34,999	187	16.0
$35,000–$49,999	140	12.0
$50,000–$74,999	159	13.6
$75,000 or more	196	16.8
Highest level of education completed		
Less than high school	242	20.8
High school	362	31.1
Some college	353	30.3
Bachelor’s degree or higher	209	17.9
Race/Ethnicity		
White, Non-Hispanic	483	41.4
Black, Non-Hispanic	112	9.6
Other, Non-Hispanic	31	2.7
Hispanic	509	43.7
2+ Races, Non-Hispanic	31	2.7
Citizenship		
Born in the United States	818	70.2
Gender		
Female	653	56.0
Employment status		
Working as a paid employee	469	40.2
Self-employed	91	7.8
On temporary layoff	22	1.9
Unemployed, but looking	162	13.9
Retired	124	10.6
Not working—disabled	145	12.4
Not working—other	153	13.1
Language spoken at home		
English	829	72.00
Spanish	311	27
Other	12	1.0

### 2.1. Assessing the Clustering Solution

We determined that there were three distinct clusters, with 777, 226, and 163 members, respectively. The *R*^2^ for this solution was 0.28. The first step in assessing the clustering solution was to look at the distribution of clustering variables (here, PHEP outcomes) between the three groups. As seen in Table 2, there were important differences between the groups, reflected in their names. The “Disengaged Skeptics” cluster contained 777 individuals, or 67% of the sample. The “Informed Unconvinced” cluster contained 226 individuals (19% of the sample). The “Open to Persuasion” cluster contained 163 individuals (14% of the sample). These names reflect attitudes towards the H1N1 vaccination. Intention to receive the H1N1 vaccination varied greatly across groups. Approximately 10% of the Disengaged Skeptics, 21% of the Informed Unconvinced, and 37% of the Open to Persuasion reported intention to get the vaccine. Post-hoc comparisons provide additional details. Compared to the Disengaged Skeptics cluster, the Informed Unconvinced cluster had greater odds of reporting that they will get the vaccine, but haven’t tried yet (OR = 2.447, 95% CI: 1.503–3.984), higher odds of reporting that they tried, but it wasn’t available (OR = 4.495, 95% CI: 2.320–8.708), and higher odds of reporting that they don’t know (OR = 1.765, 95% CI: 1.249–2.494) *vs.* reporting that they will not get the H1N1 vaccine. Compared to the Disengaged Skeptics cluster, the Open to Persuasion cluster had greater odds of reporting that they will get the vaccine, but haven’t tried yet (OR = 6.272, 95% CI: 3.881–10.135), higher odds of reporting that they have tried, but it wasn’t available (OR = 7.361, 95% CI: 3.654–14.381), and higher odds of reporting that they don’t know (OR = 1.966, 95% CI: 1.279–3.022) *vs.* reporting that they will not get the H1N1 vaccine.

**Table 2:** Distribution of PHEP outcomes between the cluster groups (*n* = 1166).

PHEP Outcomes	Disengaged Skeptics (*n* = 777) (%)	Informed Unconvinced Cluster (*n* = 226) (%)	Open to Persuasion Cluster (*n* = 163) (%)	*p*-value
Intention to get [the H1N1] vaccine?				<0.0001
Will get the vaccine but have not tried yet	7.5	13.3	26.3	
Have tried to get the vaccine but it has not been available	2.6	8.4	10.6	
Will not get the vaccine	66.0	47.8	36.9	
Don’t know	23.9	30.5	26.3	
Seasonal flu vaccination status				<0.0001
Received	0.3	100	30.1	
Intention to receive seasonal flu vaccine among those who have not received it (*n* = 889)				<0.0001
Will get the vaccine but have not tried yet/Have tried to get the vaccine but it has not been available	10.5	n/a	33.3	
Will not get the vaccine	67.2	n/a	44.7	
Don’t know	22.3	n/a	21.9	
Correct answers: knowledge regarding transmission				0.3570
0	10.9	9.7	8.0	
1	47.8	50.9	43.6	
2	41.3	39.4	48.5	
Correct answers: knowledge regarding symptoms				0.0054
0	2.8	0.4	1.8	
1	12.1	6.2	6.8	
2	19.7	19.0	14.7	
3	65.4	74.3	76.7	
Risk perception: Likelihood of getting sick from H1N1 in the next 12 months, scale from 0 (not at all likely) to 10 (very likely) *				0.0113
Mean (SD)	3.3 (2.3)	3.3 (1.9)	3.9 (2.4)	
Perceived safety of vaccine for influenza H1N1 for most people, scale from 0 (not at all safe) to 10 (very safe) *				0.0001
Mean (SD)	5.5 (2.4)	6.2 (2.1)	6.0 (2.5)	
Engagement in correct preventive behaviors				<0.0001
1	100	100	0	
2	0	0	92.0	
3	0	0	8.0	

* ANOVA used for comparisons.

There were dramatic differences in seasonal flu vaccine receipt between the three groups. All individuals in the Informed Unconvinced group had received the seasonal flu vaccine *versus* 30% of the Open to Persuasion group and 0.3% of the Disengaged Skeptics group. This difference was statistically significant (*p* < 0.0001). The groups did not differ significantly in knowledge about H1N1 transmission, but did vary in terms of knowledge regarding symptoms, risk perception, and perception of vaccine safety.

### 2.2. Uncertainty about Intention to Receive the H1N1 Vaccine

We explored the reasons behind reports that respondents “will not or do not know” if they will get the H1N1 vaccine. As seen in Table 3, a range of 15 potential reasons was presented to respondents. For the Disengaged Skeptics cluster, the top reason was not perceiving a risk of getting a serious case of influenza H1N1 (19%), followed by not being in a priority group to receive the vaccine (15%), and concern about side effects (13%). For the Informed Unconvinced cluster, the top reason was not being in a priority group to receive the vaccine (27%), followed by not perceiving a risk of getting a serious case of influenza H1N1 (17%), and followed by concern about side effects (12%). For the Open to Persuasion Cluster, the top reason was concern about side effects (26%), a lack of trust in public health officials to provide correct safety information for the vaccine (14%), and concern about getting influenza H1N1 from the vaccine (10%).

**Table 3:** Distribution (%) of main reason for responding they “will not or do not know” if they will get the H1N1 vaccine (*n* = 954).

Reasons	Disengaged Skeptics Cluster Subset (*n* = 678)	Informed Unconvinced Cluster Subset (*n* = 175)	Open to Persuasion Cluster subset (*n* = 101)	*p*-value
				<0.0001
You are not in one of the priority groups to receive the vaccine	15.2	27.4	8.9	
You have tried to get the vaccine, but it has not been available, and you will not try again	1.6	4.6	1.0	
You don’t think you are at risk of getting a serious case of influenza H1N1	19.2	16.6	8.9	
You don’t think the vaccine would be effective in preventing you from getting influenza H1N1	7.4	2.9	5.9	
You are concerned about getting influenza H1N1 from the vaccine	5.6	4.0	9.9	
You are concerned about getting another serious illness from the vaccine	4.9	4.6	2.00	
You are concerned about getting other kinds of side effects from the vaccine	13.4	12.0	25.7	
It would be too expensive for you to get the vaccine	2.8	5.7	5.9	
You don’t like shots or injections	0.7	0	2.0	
It will be too hard to get to a place where you could get the vaccine	7.8	2.9	4.0	
If you get influenza H1N1, you can get medication to treat it	2.4	0	2.0	
Your healthcare provider has told you that you shouldn’t get the vaccine	1.3	4.6	3.0	
You have been vaccinated for the seasonal flu and you believe this vaccine will also prevent you from getting H1N1	0.2	1.7	3.0	
You don’t trust public health officials to provide correct information about the safety of the vaccine	11.4	10.3	13.9	
You do not know where to get the H1N1 vaccine	6.2	2.9	4.0	

### 2.3. Communication Outcomes

Communication outcomes are key in the theoretical framework. As seen in Table 4, there were important differences in media use. Overall, there were significant differences between clusters in use of newspapers, national TV news, and local TV news. The Informed Unconvinced cluster had the highest reports of use across these categories. When asked about the source from which the most information was received about the H1N1 outbreak, all three clusters reported local television news as the top source, followed by national network television news. Further post-hoc comparisons highlighted a few significant differences. Compared to the Disengaged Skeptics cluster, the Informed Unconvinced cluster reported higher odds of reporting primary H1N1 sources of national network television news (OR = 1.978, 95% CI: 1.339–2.921) and doctor, nurse, or other medical professional (OR = 1.740, 95% CI: 1.018–2.976) *vs.* local television news. Compared to the Disengaged Skeptics cluster, the Open to Persuasion cluster had higher odds of reporting a primary H1N1 source of national network television news (OR = 1.642, 95% CI: 1.033–2.610) *vs.* local television news. The three clusters had significant differences in trust in their primary H1N1 information source, attention to news on the H1N1 outbreak, and Internet access.

**Table 4:** Information access, use, and trust, by cluster (*n* = 1166).

Characteristics	Disengaged Skeptics Cluster (*n* = 777) (%)	Informed Unconvinced Cluster (*n* = 226) (%)	Open to Persuasion Cluster (*n* = 163) (%)	*p*-value
Number of days in past week… (mean, SD) *				
Read a newspaper	1.5 (2.4)	2.5 (2.7)	1.5 (2.1)	<0.0001
Watched the national news on television	2.8 (2.7)	3.9 (2.8)	3.8 (2.8)	<0.0001
Watched the local news on television	3.4 (2.7)	4.7 (2.5)	4.4 (2.6)	<0.0001
Read news on the Internet	1.9 (2.6)	2.0 (2.6)	1.7 (2.4)	0.4396
Source from which most information was received about the H1N1 outbreak				0.0013
Local television news	43.3	36.9	36.2	
National network television news	16.1	27.1	22.1	
Cable network television news station	4.3	2.7	6.8	
Non-English speaking television station	3.0	1.8	3.7	
National newspaper	0.5	0.4	0.6	
Local newspaper	4.8	6.7	1.2	
Radio	5.5	4.9	4.3	
Internet	9.7	6.7	6.8	
Family member or friend	5.6	2.2	4.3	
Doctor, nurse or other medical professional	7.2	10.7	14.1	
Trust in source from which most H1N1 information was received				0.0011
Reported trust	53.2	64.2	65.0	
Level of attention paid to the news on H1N1 outbreak, scale of 0 (no attention) to 10 (a lot of attention) *				<0.0001
Mean (SD)	5.7 (2.5)	6.2 (2.1)	7.4 (2.5)	
Access to the Internet	58.9	62.4	37.4	<0.0001
Have a social networking profile				0.0234
Yes	42.8	42.2	31.3	

* ANOVA tests for three-way comparisons of means.

### 2.4. Information-Seeking

Another substantive area of interest in the theoretical model is information-seeking. The Open to Persuasion cluster had the highest rates of information-seeking about H1N1 (36% *vs.* 13% of the Informed Unconvinced cluster and 11% of the Disengaged Skeptics cluster). Among the 174 individuals who reported information-seeking, there were no significant differences in reports of three key challenges: effort to find the information needed, frustration felt during the search, or concern about the quality of the information found. However, the Informed Unconvinced cluster had much higher rates of reporting that the information found was too hard to understand (30% *vs.* 12% of the Open to Persuasion cluster and 6% of the Disengaged Skeptics cluster, *p* < 0.01). Overall, the three groups had high rates of confidence in finding advice or information about H1N1 if needed, ranging from 67%–79%. The differences were not statistically significant.

### 2.5. Social Determinants

The final phase of inquiry focused on the link between social determinants and cluster membership. As seen in Table 5, there were a few important differences between the clusters. The groups differed significantly by income level (*p* < 0.01). The median income group for the Disengaged Skeptics cluster was $20,000–$34,999, $35,000–$49,999 for the Informed Unconvinced Cluster, and less than $20,000 for the Open to Persuasion Cluster. Post-hoc comparisons reveal that compared to the Disengaged Skeptics cluster, the Informed Unconvinced cluster had greater odds of reporting the highest income bracket (OR = 1.524, 95% CI: 1.015–2.288) *vs.* the lowest income bracket. Compared to the Disengaged Skeptics cluster, the Open to Persuasion cluster had lower odds of reporting the highest income bracket (OR = 0.236, 95% CI: 0.115–0.482) *vs.* the lowest income bracket. The three clusters had significantly different racial/ethnic profiles (*p* = 0.0003). Over half (57%) of the Open to Persuasion Cluster were Hispanic and over one-quarter (28%) were White, Non-Hispanic. Within the Informed Unconvinced Cluster, over half (53%) were White, Non-Hispanic, and about one-third (33%) were Hispanic. The Disengaged Skeptics cluster had 41% of its members identifying as White, Non-Hispanic and 44% as Hispanic. Compared to the Disengaged Skeptics cluster, the Informed Unconvinced cluster had lower odds of identifying as Hispanic (OR = 0.590, 95% CI: 0.425, 0.817) *vs.* White, non-Hispanic. Compared to the Disengaged Skeptics cluster, the Open to Persuasion cluster had greater odds of identifying as Other, non-Hispanic (OR = 2.612, 95% CI: 1.040–6.561) and greater odds of identifying as Hispanic (OR = 1.933, 95% CI: 1.313–2.848) *vs.* White, non-Hispanic. In terms of language spoken at home, the Open to Persuasion cluster had the highest proportion of members reporting Spanish as the predominant language (48%) *versus* 14% of the Informed Unconvinced and 27% of the Disengaged Skeptics clusters. The difference was statistically significant (*p* < 0.01).

**Table 5:** Pattern of social determinants between three cluster groups (*n* = 1166).

Social Determinants	Disengaged Skeptics Cluster (*n* = 777) (%)	Informed Unconvinced Cluster (*n* = 226) (%)	Open to Persuasion Cluster (*n* = 163) (%)	*p*-value
Income ($)				0.0003
Less than 20,000	40.8	34.5	54.6	
20,000–34,999	16.9	14.2	14.7	
35,000–49,999	11.5	14.2	11.7	
50,000–74,999	13.4	14.6	13.5	
75,000 +	17.5	22.6	5.5	
Highest level of education completed				0.1899
Less than high school	20.9	17.3	25.2	
High school	30.2	30.1	36.2	
Some college	30.9	32.3	24.5	
Bachelor’s degree or higher	18.0	20.4	14.1	
Race/ethnicity				0.0003
White, Non-Hispanic	41.1	52.7	27.6	
Black, Non-Hispanic	10.0	9.3	8.0	
Other, Non-Hispanic	2.5	2.2	4.3	
Hispanic	43.9	33.2	57.1	
2+ Races, Non-Hispanic	2.6	2.7	3.1	
Born in the United States				<0.0001
Yes	69.5	85.4	52.2	
Language usually spoken at home				<0.0001
English	72.3	86.0	51.2	
Spanish	26.6	13.5	47.5	
Other	1.2	0.5	1.2	
Gender				0.0022
Female	52.4	63.7	62.6	
Work status				<0.0001
Working as a paid employee	43.2	37.6	29.5	
Self-employed	9.0	5.3	5.5	
On temporary layoff	2.3	0.4	1.8	
Unemployed, but looking	15.4	8.9	13.5	
Retired	6.2	25.7	11.0	
Not working—disabled	10.3	13.7	20.9	
Not working—other	13.5	8.4	17.8	
Age group (years)				<0.0001
18–24	12.7	4.9	6.1	
25–34	24.7	14.6	22.1	
35–44	22.3	16.4	25.2	
45–54	21.4	17.7	15.3	
55–64	13.1	20.4	16.6	
65–74	5.0	18.6	13.5	
75+	0.8	7.5	1.2	
Marital status				0.0107
Married	42.9	50.4	50.3	
Widowed	3.1	6.7	3.7	
Divorced	9.8	11.1	11.0	
Separated	4.3	5.3	5.5	
Never married	28.2	17.7	17.8	
Living with partner	11.8	8.9	11.7	

The Informed Unconvinced cluster had the highest percentage of individuals born in the United States, with significant differences between groups. Table 5 highlights the patterning of employment status between clusters. Compared to the Disengaged Skeptics cluster, the Informed Unconvinced cluster had greater odds of reporting being retired (OR = 4.778, 95% CI: 3.045–7.497). Compared to the Disengaged Skeptics cluster, the Open to Persuasion cluster had greater odds of reporting being retired (OR = 2.625, 95% CI: 1.412–4.882), not working—disabled (OR = 2.975, 95% CI: 1.800–4.917), and not working—other (OR = 1.933, 95% CI: 1.160–3.221). The age distribution across cohorts was significantly different (*p* < 0.0001), with the Disengaged Skeptics cluster including more young people and the Informed Unconvinced cluster including many older adults.

### 2.6. Summary of the Three Clusters

The largest cluster was the Disengaged Skeptics cluster. Only about 10% of members indicated an intention to get the H1N1 vaccine. The group reported low uptake of/intention to receive the seasonal flu vaccine as well. The Disengaged Skeptics cluster reported low risk perception related to H1N1 and relatively lower perceptions of H1N1 vaccine safety. Key barriers to H1N1 vaccination included low risk perception, concern about side effects, and a lack of trust in public health officials. In terms of their information environment, this group had somewhat lower trust in their primary source of H1N1 information, lower attention to H1N1 news, and less information-seeking related to H1N1 than the other clusters. This group relied more heavily on local television news than the other groups. From a demographics standpoint, this group included more of the young respondents, included large numbers of White, non-Hispanics and Hispanics, had a lower percentage of women, and had the highest percentages of working adults compared to the other clusters.

In contrast, the Informed Unconvinced cluster was characterized by 21% indicating intention to receive the H1N1 vaccine, despite the fact that all of the individuals in this group reported receipt of the seasonal flu vaccine. As a group, they were relatively moderate in assessing risk of contracting H1N1 and vaccine safety. In addition to local and national network news, they relied on healthcare professionals as primary sources of H1N1 information. Overall, this group reported the highest media use overall, as well as Internet access. Also, among those who did not know or would not get the H1N1 vaccine, the top reason for not getting the vaccine in this cluster was not being part of a priority group. This group had the highest income levels, the largest proportion of White-non-Hispanic individuals, the highest proportion of US-born individuals, and the highest proportion of women. A large portion of the group was retired or not working for other reasons. They tended to be older as well.

The Open to Persuasion cluster was characterized by relative openness to the H1N1 vaccine; interestingly, less than one-third of this group had received the seasonal flu vaccine. Among those who had not received it, almost half said they would not get the seasonal flu vaccine. This group had lower risk perception than the other groups and more moderate perceptions about vaccine safety. They engaged in a higher number of correct preventive behaviors than the other groups. They paid a great deal of attention to H1N1-related news and had much higher rates of information-seeking than the other clusters. In addition to local and national network news, they relied on healthcare professionals as primary sources of H1N1 information. Demographically, they tended to have lower income levels and greater proportions of Hispanics, individuals from homes in which Spanish was the primary language, and individuals born outside of the United States.

## 3. Discussion

This study demonstrates the potential of using audience segmentation techniques to identify subgroups of vaccine-hesitant individuals and plan for effective engagement. We identified three important subgroups of individuals who did not receive the H1N1 vaccine, the Open to Persuasion, Informed Unconvinced, and Disengaged Skeptics clusters. The Open to Persuasion cluster seemed to be the furthest along the continuum towards vaccine acceptance for H1N1. There may have been an important opportunity to address the needs of this group and increase vaccination rates. By providing details about the group’s current beliefs, behaviors, and communication profiles, the analysis is a useful starting point for targeted engagement. The Informed Unconvinced cluster appeared to be in the middle along the continuum and may have been relatively sophisticated consumers of health information. The issue may be that they were discriminating consumers of information and the information they received either did not satisfy them or did not prompt them to accept H1N1 vaccination. The communication strategies targeting such a group may need to address a high level of knowledge and potential skepticism related to this disease and vaccine specifically. Finally, the Disengaged Skeptics cluster appeared to be relatively far away from acceptance of the H1N1 vaccine. This group may have needed to be brought along slowly regarding more than just the H1N1 vaccine. The statistically significant differences between clusters on a number of key theoretical constructs in our framework support the validity of the groupings that emerged from this segmentation analysis. The dramatic differences between groups in seasonal influenza vaccination status are an example of a result that would prompt further inquiry to replicate or explain as part of the development of customized communication strategies. In line with the SIM, our findings highlight the critical roles played by communication and information in vaccination decisions, which can provide an opening to engaging with the diverse segments of the public along the vaccine hesitancy continuum for a given vaccine. Use of a health behavior or health communication theory to drive the segmentation analysis allows for effective targeting of key variables to promote behavior change [27].

The results of this study highlight the potential to use this approach to develop communication strategies to target clusters of vaccine-hesitant individuals. As seen by Leask *et al.*’s [30] synthesis of studies related to parental vaccine hesitancy, the field will benefit from integration of multiple segmentation efforts. This will yield both estimates of group sizes as well as useful classification criteria. It may be particularly important to assess the size and characteristics of dissonant groups, which may be small, but have strong impact if their messages are amplified greatly by interpersonal channels, online and offline. At the same time, the literature includes a number of assessments linking vaccine uptake to clustering by social networks, geography, sociodemographic characteristics, and psychosocial characteristics [13,31,32]. The addition of the social determinants and communication outcomes extends the ability of public health practitioners to segment the market more effectively.

Targeted strategies that account for different information consumption and source preference patterns are likely required. This is particularly important as the media environment becomes increasingly fragmented [33] and individuals are less likely to encounter information that is contrary to the existing beliefs in their typical media platforms and interpersonal networks. Primary sources for information about new diseases/vaccines may differ widely in their ability to mitigate concerns about safety and encourage sufficient attention and concern [19]. A recent surveillance study that assessed web-based information about vaccines found that over two-thirds (69%) of the content of articles, blogs, reports, *etc.* were positive, but the remaining one-third of the content had a negative tone and emphasized beliefs that could have tremendous influence on the public [34]. In addition to strategic methods of reaching diverse clusters of the vaccine-hesitant individuals, it is vital to ensure that communications focus on engagement and informed decision-making. It is increasingly clear that top-down, authoritative communication strategies do not support public health goals and an engaged approach, whether on the part of governments, healthcare professionals, or community organizations, can support open discussions and decision-making [22].

Taking a social ecological perspective, we recognize that vaccination decisions are not made in a vacuum, but instead are influenced by (and influence) factors at the individual, interpersonal, community, organizational, political, and cultural levels [35,36]. This prompts attention to the ways in which individuals engage with information and interact with social groups and networks (online and offline), communities, institutions (particularly those in the healthcare sector as well as employers), policies (including prioritization of population groups), and cultural drivers. A segmentation analysis such as this can therefore play an important role in a broader assessment, such as the World Health Organization’s Tailoring Immunizations Program. This program guides member states in the conduct of multi-level assessments to promote vaccination by identifying vulnerable populations (using segmentation), assessing barriers to vaccination (both supply-side and demand-side), and creating effective responses [37]. The segmentation variables presented here could serve as a complement to existing suggestions for segmentation analysis and further refine the ability to identify and engage with key subgroups. Once more, a theory-driven set of variables is useful as it supports a targeted selection of variables (both for identifying subgroups and also for creating response strategies).

The audience segmentation analysis demonstrated here may be useful in a range of settings. This assessment was conducted at the national level, but the approach could be applied at local or regional levels as well. Fielding the survey was both cost-effective and efficient with the use of an online panel. This mode of survey administration may not be appropriate if the panels do not sufficiently represent vulnerable populations. For this study, we addressed this challenge by oversampling key racial/ethnic minority groups and individuals living under the United States Federal Poverty Level. Regardless of mode of administration, this type of survey can be fielded in much the same way as any other quantitative survey and can be utilized in settings where that method is appropriate and feasible. The data analysis required is fairly straightforward, though it requires familiarity with cluster analysis. Despite the potential of the segmentation approach, an important challenge relates to the ability to conduct segmentation in real-time. While theories of behavior change provide a useful starting point, the time requirements of customizing the set of segmentation variables and behavioral antecedents for a given vaccine pose a challenge. Also, even in cases where automated approaches are appropriate and the survey can be administered quickly, time is needed to create intervention and communication strategies based on those data. There may be an opportunity to integrate rapid segmentation assessments with ongoing monitoring efforts, such as programs conducting ongoing surveillance of vaccine-related discussions on social media [22]. A useful example of the application of this approach comes from the field of climate change. A group of scientists have segmented the American population into the “Six Americas” of climate change and have characterized the segments in terms of beliefs, concern, and engagement about climate change. They have also tracked the changes in the sizes of the segments over time and studied their policy and communication preferences. In this way, appropriately directed and tailored engagement strategies can be created for diverse subgroups [38,39].

As with any study, the results must be interpreted in the context of a set of limitations. First, the clustering solution presented only explains 28% of the variation in the model. This is a function of our decision to focus on PHEP outcomes to segment the population. The benefit of our approach is that the resulting behavior-driven clusters can then be tied back to potential communication leverage points to support behavior change. In addition to the areas typically focused on (sociodemographic factors, geographic variation, *etc.*), understanding groups’ channel preferences, trusted sources, and other communication-related details allows for much more sophisticated engagement and provision of information. Second, we did not assess three of the components of the model (social networks, information processing, and place) in this study, which should be added in future work. Third, the timing of the survey (March 2010, as the 2009–2010 influenza season was winding down) impacts the questions regarding intention to receive the H1N1 vaccine, so we interpreted them in the context of responses regarding attempts to receive the H1N1 vaccine, reasons for being unsure about H1N1 vaccination, and seasonal influenza vaccine receipt. The strengths of the study outweigh these limitations. The use of a theory-driven strategy for segmentation provides insight into communication/engagement leverage points that may be particularly useful in the context of vaccines with which the public is unfamiliar. The guidance from the SIM model prompted inclusion of key segmenting variables, while maintaining parsimony. Another strength of the study is the use of cluster analysis without *a priori* specification of the number of clusters. This data-driven approach allowed for cluster patterns to emerge systematically from the data.

Further research efforts to use this approach to inform targeted public engagement strategies may unearth additional sub-groupings among the vaccine-hesitant. Additional segmentation studies focused on a range of vaccines will also allow for deeper understanding of the common and vaccine-specific aspects of hesitancy. By better understanding the spectrum of vaccine-hesitancy, we can begin to put into place the necessary supports to inform and support health-promotive decision-making among the public.

## 4. Materials and Methods

Data come from a US-based survey focused on the 2009 H1N1 epidemic, fielded in March 2010.

### 4.1. Respondents

We collected data from respondents drawn from a nationally representative sample of U.S. adults age 18 and older, participating in Knowledge Networks’ KnowledgePanel^©^. Members of this panel are recruited using a dual sampling frame, a combination of Random Digital Dial and Address-Based Sampling, which allows for sampling of individuals with no telephone land lines. Additionally, when recruited, non-Internet households are provided with a laptop computer and free Internet access. Participants received nominal cash incentives to participate in this web-based survey. For the current study, participants from minority ethnic/racial groups and those living under the United States Federal Poverty Level were oversampled. All research procedures were approved by the Institutional Review Board of the Harvard School of Public Health.

### 4.2. Measures

The survey questions were constructed based on two main sources: focus group data and pre-existing surveys. We conducted five focus groups with 46 participants from diverse ethnic/racial and socioeconomic backgrounds. Key themes included H1N1 knowledge; preventive behavior, attitudes, beliefs; mass and interpersonal communication; and emergency preparedness in general. This information was used to generate new survey items, which were combined with items adapted from the Harvard Opinion Research Program H1N1 Survey [18], the Health Information National Trends Survey (HINTS) [40], and the CDC’s Behavioral Risk Factor Surveillance System (BRFSS) [41]. The survey was finalized after a round of cognitive interviews with individuals similar to the target population.

#### 4.2.1. PHEP Outcomes

The theoretical framework drove selection of the clustering variables, those in the “PHEP outcomes” portion of the framework. These variables included: *Knowledge of H1N1 transmission* (To the best of your knowledge, how can someone get H1N1? Response options: from being in close contact with someone who has H1N1 (within arm’s length of someone); from eating pork; from coming in contact with pigs; and from touching objects (*i.e.*, glass) recently touched by someone with flu). *Risk perception* (Using a scale of 0 (not at all likely) to 10 (very likely), select a number that is closest to the statement: How likely do you think it is that you may get sick from H1N1 during the next 12 months?) *Safety of vaccine* (On a scale of 0 (not at all safe) to 10 (very safe), how safe do you believe the vaccine for influenza H1N1 will generally be for most people to take?) *Protective behavior against H1N1* (In response to news reports of H1N1, have you done any of the following? Response options: sought out a vaccine for you or your loved ones; avoided places where many people are gathered together, like sporting events, malls, or public transportation; reduced human contact with people outside of your immediate family such as signs of affection (hug/kiss), shaking hands, or sign of peace during worship; talked with your doctor about health issues related to H1N1; worn a face mask; washed your hands or used hand sanitizer more frequently; began coughing with your mouth covered; avoided people you think may have recently visited Mexico; avoided Mexican restaurants or grocery stores; avoided eating pork products; gotten a prescription for or purchased anti-virals, such as Tamiflu or Relenza without symptoms of illness; avoided air travel; avoided mass transit such as buses and trains; stayed home; and kept your children home from school). The count of correct responses was utilized for the analysis. *Behavior: seasonal flu vaccine receipt* (Have you received the seasonal flu vaccine this flu season? Response options: yes or no). For those who had not received the vaccine, a follow-up question was asked (Do you think you will or will not get this vaccine? Response options: yes, I will get the vaccine but have not tried yet; yes, I have tried to get the vaccine but it has not been available; no, I will not get the vaccine; and don’t know). The first two responses were combined for the creation of the variable used in the clustering algorithm. In addition to the clustering variables, we also asked about respondents’ *intention to receive the H1N1 vaccination:* (Do you think you will or will not get (the H1N1) vaccine? Response options: yes, I will get the vaccine but have not tried yet; yes, I have tried to get the vaccine but it has not been available; no, I will not get the vaccine; and don’t know).

#### 4.2.2. Communication Variables

To assess the link between communication outcomes and clusters, we utilized the following variables: *Main source of information* (From what source have you received the most information about the H1N1 outbreak? Response options: local television news; national network television news, such as ABC World News, CBS Evening News, NBC Nightly News, or Fox News; cable network television news station, such as CNN or MSNBC; non-English speaking television station; national newspaper, such as the *New York Times*, *Wall Street Journal*, or *USA Today*; local newspaper; non-English newspaper; radio; Internet; family member or friend; and doctor, nurse or other medical professional). *Media exposure* (In the past seven days, how many days did you… Response options: read a newspaper; watch the national news on television; watch the local news on television; read news on the Internet). *Attention to H1N1 news* (In general, how much attention have you paid to the news on H1N1 outbreak. On a scale of 0–10 where “0” is no attention and “10” is a lot of attention, how much attention have you paid?). *Social media usage* (Do you currently have your own profile on a social networking site like MySpace, Facebook, or Twitter? Response options: yes or no.).

#### 4.2.3. Information-Seeking

We utilized a set of variables to delve into information-seeking patterns. *Information seeking* (Have you actively searched for information about H1N1? Response options: yes or no. If yes, where did you go for more information during your last search? Response options: local television news; national network television news, such as ABC World News, CBS Evening News, NBC Nightly News, or Fox News; cable network television news station, such as CNN or MSNBC; non-English speaking television station; national newspaper, such as the *New York Times*, *Wall Street Journal*, or USA Today; local newspaper; non-English newspaper; radio; Internet; family member or friend; medical professional). *Barriers to searching* (Based on the results of your most recent search for information on H1N1, how much do you agree or disagree with the following statements? Response options: strongly agree, somewhat agree, somewhat disagree, strongly disagree. Statements: it took a lot of effort to find the information you needed; you felt frustrated during your search; you were concerned about the quality of the information; and the information you found was too hard to understand). *Confidence in finding information* (Overall, how confident are you that you could get advice or information about H1N1 if you needed it? Response options: completely confident; very confident; somewhat confident; a little confident; and not confident at all). *Trust of primary information source* (Thinking about the source you have received the most H1N1 information from, [would you say that you] trust information from that source? Response options: yes or no).

#### 4.2.4. Additional Variables

*Reasons for not getting vaccine* (If you don’t think or don’t know whether you will get the H1N1 vaccine, what is the main reason for your decision? Response options: you are not in one of the priority groups to receive the vaccine; you have tried to get the vaccine but it has not been available and you will not try again; you don’t think you are at risk of getting a serious case of influenza H1N1; you don’t think the vaccine would be effective in preventing you from getting influenza H1N1; you are concerned about getting influenza H1N1 from the vaccine; you are concerned about getting another serious illness from the vaccine; you are concerned about getting other kinds of side effects from the vaccine; it would be too expensive for you to get the vaccine; it will be too hard to get to a place where you could get the vaccine; you don’t like shots or injections; if you get influenza H1N1, you can get medication to treat it; your healthcare provider has told you that you shouldn’t get the vaccine; you have been vaccinated for the seasonal flu and you believe this vaccine will also protect you from getting H1N1; you don’t trust public health officials to provide correct information about the safety of the vaccine; and you do not know where to get the H1N1 vaccine).

#### 4.2.5. Socioeconomic Factors and Demographic Attributes

Finally, to assess the link between social determinants of health and clusters, we utilized the following variables: *Household income* (What was the total combined income of your family in 2009, including income from all sources such as wages, salaries, Social Security or retirement benefits, help from relatives and so forth? Please tell us the total income before taxes. Categories created from response options: less than $20,000; $20,000–$34,999; $35,000–$49,999; $50,000–$74,999; and $75,000 or more). *Education* (What is the highest level of school you completed? Categories created from response options: less than high school; high school; some college; and bachelor’s degree or higher). *Race/ethnicity* (What race do you consider yourself? AND how would you describe yourself? Are you Hispanic or Latino?) Categories created from response options: White, Non-Hispanic; Black, Non-Hispanic; other, Non-Hispanic; Hispanic; 2+ Races, Non-Hispanic.). *Nativity* (Were you born in the United States? Response options: yes or no). *Gender* (Response options: male or female). *Employment status* (What is your work situation? Categories created from response options: working as a paid employee; working—self-employed; on temporary layoff; unemployed, but looking; retired; disabled; and not working—other).

*Age* (We asked age in years and created the following categories from response options: 18–24; 25–34; 35–44; 45–54; 55–64; 65–74; and 75+). *Marital status* (Which of the following best describes your current marital status? Categories created from response options: married; widowed; divorced; separated; never married; and living with partner). *Place* (The intake form assessed Metropolitan Statistical Area. Response options: Metro and non-metro.) *Language* (What language do you usually speak at home? Categories created from response options: English; Spanish; and other).

### 4.3. Data Analysis

For this analysis, we excluded individuals who had received the H1N1 vaccine at the time of the survey (*n* = 356) and those with incomplete data for the clustering variables (*n* = 47) to yield a sample of 1166 individuals. Data were analyzed using SAS v 9.4 [42]. Cluster analysis is an established method for conducting audience segmentation analyses [26]; we employed agglomerative hierarchical cluster analysis to cluster respondents [43]. The clustering variables (seasonal flu vaccination status, intention to receive seasonal flu vaccination among those who had not received it, knowledge of H1N1 transmission, knowledge of H1N1 symptoms, protective behaviors, perceived likelihood of contracting H1N1, perception of H1N1 vaccine safety) were transformed into a standardized, squared Euclidian distance matrix. We employed Ward’s method [44], a clustering algorithm that is particularly well-suited to yielding similar group sizes and facilitating comparison of determinants between identified clusters [45,46]. Following established procedures [47], we determined the number of clusters by examining the dendogram and evaluating the Pseudo F (PSF) and Pseudo t^2^ (PSt^2^) values for the full range of cluster solutions generated. Most comparisons by cluster were conducted using chi-square analyses. The exceptions were: (a) instances in which the cells had such small expected values that chi-square analyses were inappropriate and we used Fisher’s exact test instead and (b) instances in which we were comparing means and we utilized ANOVA procedures instead. These exceptions have been marked in the tables. Post-hoc comparisons were conducted using logistic regression procedures for outcomes with more than two response levels.

## 5. Conclusions

As demonstrated here, audience segmentation analysis can be useful to unpack heterogeneity among the vaccine-hesitant. This systematic approach identifies key audience segments and creates profiles of their sociodemographic, psychosocial, and communication patterns. Such analyses can inform the development of tailored public engagement efforts, potentially increasing the efficiency and effectiveness of efforts to promote large-scale vaccine uptake.
